# Multilevel auditory and cognitive processing in post-stroke aphasia: associations with language performance using ABR, LLR, and P300: a cross-sectional observational study

**DOI:** 10.1590/1516-3180.2026.3635.08042026

**Published:** 2026-07-20

**Authors:** Agit Şimşek, Nihal Sümeyye Ulutaş, Feyza Deniz Saman

**Affiliations:** I Department of Speech and Language Therapy, Faculty of Health Sciences, Inönü University, Malatya, Türkiye.; II Faculty of Medicine, Turgut Özal University, Ankara, Türkiye.; III Independent Researcher, Malatya, Türkiye.

**Keywords:** Aphasia, Stroke, Evoked Potentials, Auditory, Evoked Potentials, Auditory, Brain Stem, Event-Related Potentials, P300, Post-Stroke Aphasia, Auditory Processing, Late Latency Responses, Cognitive Processing Speed, Language Performance, Speech and Language Therapy

## Abstract

**BACKGROUND:**

Aphasia following stroke is primarily characterized by language impairment; however, accumulating evidence suggests that deficits in auditory and cognitive processing may also contribute to impaired language function. Auditory evoked potentials provide objective markers of neural processing across subcortical, cortical, and cognitive levels and may help clarify the neurophysiological mechanisms underlying post-stroke aphasia.

**OBJECTIVES:**

To investigate multilevel auditory processing and its relationship with language performance in individuals with post-stroke aphasia using Auditory Brainstem Responses (ABR), Late Latency Responses (LLR), and P300 potentials.

**DESIGN AND SETTING:**

Cross-sectional observational study conducted at a university hospital in Türkiye.

**METHODS:**

Twenty-nine individuals with post-stroke aphasia and 33 age-matched healthy controls were included. Language performance was assessed using the Aphasia Language Assessment Test and a standardized naming task. Auditory processing was evaluated using ABR, LLR (P1, N1, P2, N2), and P300 potentials recorded according to standard electrophysiological protocols. Group comparisons were performed for latency and amplitude measures, and correlation analyses were conducted to examine associations between electrophysiological parameters and language performance.

**RESULTS:**

Compared with healthy controls, individuals with aphasia demonstrated significantly prolonged latencies in ABR waves and interpeak intervals, as well as in all LLR and P300 components (p < 0.05). No significant group differences were observed in amplitude measures. Naming performance was significantly lower in the aphasia group. Although correlations between language performance and electrophysiological measures did not reach statistical significance, moderate negative trends were observed between naming scores and N2 and P300 latencies.

**CONCLUSION:**

Post-stroke aphasia is associated with delayed auditory processing across subcortical, cortical, and cognitive levels, reflecting generalized slowing rather than reduced neural recruitment. Latency-based auditory evoked potential measures may complement behavioral language assessments and support a multilevel auditory–cognitive framework for aphasia evaluation and rehabilitation.

## INTRODUCTION

Post-stroke aphasia is among the most common and disabling outcomes of cerebrovascular accidents, affecting approximately one-third of stroke survivors and substantially impairing communication and quality of life.^1^ Although aphasia has traditionally been conceptualized as a disorder of language production and comprehension, growing evidence indicates that it also involves impairments in auditory processing, attention, and cognitive control mechanisms that support functional language use.^2,3^ From a speech and language therapy perspective, this broader framework highlights the importance of integrating linguistic and neurofunctional approaches in aphasia assessment.

Language performance, particularly naming ability, is a core component of clinical aphasia evaluation and a sensitive indicator of impairment severity and recovery.^4^ Naming tasks require coordinated auditory perception, lexical access, semantic processing, and executive control, making them vulnerable to disruption across multiple neural levels. Although behavioral language assessments provide essential information regarding functional outcomes, they may not fully capture the neural mechanisms underlying impaired language performance, emphasizing the need for complementary objective measures.

Auditory evoked potentials (AEPs) provide a non-invasive and temporally precise method for examining neural processing along the auditory pathway. Auditory Brainstem Responses (ABR) reflect early subcortical auditory transmission, Late Latency Responses (LLR) index cortical auditory detection and discrimination, and the P300 component is associated with attention allocation and working memory updating.^5^ Previous aphasia studies have primarily focused on isolated cortical components and consistently reported prolonged latencies, suggesting slowed auditory and cognitive processing.^3^ However, amplitude findings remain inconsistent, and subcortical auditory processing has received limited attention. Although ABR is not routinely included in aphasia assessment, early auditory timing may influence the quality of auditory input available for higher-level language processing.

Emerging evidence suggests that early auditory processing may also be impaired in post-stroke aphasia. Mourad et al.^6^ reported delayed auditory brainstem responses in individuals with aphasia, raising the possibility that inefficiencies at early auditory stages contribute to downstream cortical and cognitive processing delays. Despite this theoretical and clinical relevance, few studies have examined brainstem, cortical, and cognitive auditory responses within the same cohort, limiting integrative interpretations of auditory–language interactions.

Accordingly, this study aimed to investigate language performance—particularly naming ability—and auditory evoked potentials across multiple neural levels (ABR, LLR, and P300) in individuals with post-stroke aphasia compared with age- and sex-matched healthy controls. We hypothesized that individuals with aphasia would exhibit prolonged electrophysiological latencies reflecting slowed auditory and cognitive processing and that these delays would be associated with reduced language performance. By integrating behavioral and electrophysiological measures, this study aims to contribute to objective multilevel assessment frameworks in speech and language therapy.

## OBJECTIVE

To investigate auditory and cognitive processing across multiple neural levels and examine their association with language performance in individuals with post-stroke aphasia using ABR, LLR, and P300 event-related potentials.

## METHODS

### Study design and participants

This cross-sectional observational study was conducted at İnönü University. The study included 29 individuals with post-stroke aphasia and 33 age- and sex-matched neurologically healthy controls. Aphasia diagnosis was established by a speech and language therapist based on clinical evaluation and standardized language assessment. Inclusion criteria for the aphasia group were: (i) history of ischemic or hemorrhagic stroke, (ii) aphasia diagnosis, (iii) age 18–80 years, and (iv) adequate hearing sensitivity for auditory testing. Exclusion criteria for all participants included additional neurological disorders, severe cognitive impairment, psychiatric illness, and uncorrected hearing loss. Pure-tone audiometry confirmed hearing thresholds ≤25 dB HL at 500–4,000 Hz in all participants.

### Ethical approval

The study was conducted in accordance with the Declaration of Helsinki and approved by the İnönü University Health Sciences Scientific Research Ethics Committee (Approval No 2024/6333; Date: September 24, 2024). Written informed consent was obtained from all participants prior to participation.

### Language assessment

Language performance was assessed using the Aphasia Language Assessment Test (ADD) and a naming task. The ADD was administered according to standardized procedures to evaluate overall language ability. In the naming task, participants were asked to name visually or auditorily presented stimuli. Each correct response received one point, whereas incorrect responses received zero points. Total scores were used for statistical analyses.

### Electrophysiological recordings

Auditory evoked potentials were recorded in a sound-attenuated room using the Neurosoft Neuro Audio system. Electrode placement followed the international 10–20 system, and electrode impedance was maintained below 5 kΩ throughout testing.

**ABR:** ABR recordings were obtained using 90 dB nHL rarefaction click stimuli presented at a repetition rate of 21.1/s. Filter settings were 150–3,000 Hz. Absolute latencies of Waves I, III, and V, as well as interpeak intervals (I–III and I–V), were analyzed for both ears.

**LLR:** LLR recordings were elicited using 65 dB click stimuli presented at a repetition rate of 1.1/s with filter settings of 30–150 Hz. Latencies of the P1, N1, P2, and N2 components and peak-to-peak amplitudes (P1–N1 and P2–N2) were measured. Click stimuli were preferred to ensure temporal precision and comparability across subcortical and cortical auditory responses.

**P300 Potentials:** P300 responses were recorded using an auditory oddball paradigm with 1,000 Hz frequent and 2,000 Hz rare tones. Participants were instructed to mentally count the rare stimuli. Recordings were obtained from the Cz electrode referenced to M1–M2, with Fpz as ground. P300 latency was measured as the primary outcome. Only participants achieving ≥ 80% accuracy in the counting task were included in the analysis.

### Statistical analysis

Statistical analyses were performed using SPSS version 23.0. Data normality was assessed using the Shapiro–Wilk and Kolmogorov–Smirnov tests. Group comparisons were conducted using independent samples t-tests for normally distributed variables and Mann–Whitney U tests for non-normally distributed variables. Analysis of covariance (ANCOVA) was performed with age as a covariate. False discovery rate (FDR) correction was applied to control for multiple comparisons. Correlations between language performance and electrophysiological measures were examined using partial correlation analyses controlling for age. Statistical significance was set at p < 0.05.

## RESULTS

### Participant characteristics

A total of 62 participants were included, comprising 29 individuals with post-stroke aphasia and 33 healthy controls. Demographic characteristics of both groups are presented in **Table 1**. No statistically significant differences were observed between groups in age or sex distribution (p > 0.05).

**Table 1:** Demographic characteristics of the study groups

Variable	Aphasia group (n = 29)	Control group (n = 33)	p value
Age (years), mean ± SD	55.9 ± 7.8	53.1 ± 8.3	0.207
Sex (male/female), n	20/9	19/14	0.368

### Language performance

Language assessment results are summarized in **Table 2**. The aphasia group demonstrated significantly lower Aphasia Language Assessment Test (ADD) total scores and ADD success percentages than the control group after adjustment for age. These differences remained statistically significant following FDR correction (p < 0.001).

**Table 2:** Comparison of language and electrophysiological measures between groups (ANCOVA with age as covariate, FDR-corrected)

Measure	Aphasia group (mean ± SD/median)	Control group (mean ± SD/median)	ANCOVA F	p value	FDR-adjusted p
ADD Total Score	15.28 ± 4.96	19.67 ± 0.82	25.34	< 0.001	< 0.001
ADD Success (%)	79.66 ± 28.1	98.33 ± 4.7	18.67	< 0.001	< 0.001
Right ABR Wave III (ms)	3.85 ± 0.04	3.54 ± 0.04	12.67	< 0.001	0.002
Right ABR Wave V (ms)	6.00 ± 0.39	5.65 ± 0.28	8.92	0.005	0.009
Left ABR Wave III (ms)	3.89 ± 0.05	3.58 ± 0.03	13.45	< 0.001	0.002
Left ABR Wave V (ms)	5.93 ± 0.46	5.62 ± 0.21	7.89	0.007	0.012
P1 Latency (ms)	80.86 ± 5.7	66.84 ± 1.22	9.23	0.004	0.009
N1 Latency (ms)	128.43 ± 7.15	98.97 ± 1.88	22.18	< 0.001	< 0.001
P2 Latency (ms)	192.99 ± 6.74	120.96 ± 2.59	35.67	< 0.001	< 0.001
N2 Latency (ms)	249.15 ± 8.54	145.99 ± 2.45	42.15	< 0.001	< 0.001
P300 Latency (ms)	346.76 ± 41.3	269.72 ± 18.7	15.34	< 0.001	< 0.001

### ABR

Group comparisons of ABR latency and interpeak interval measures are presented in **Table 2**. After adjustment for age using ANCOVA and correction for multiple comparisons, the aphasia group showed significantly prolonged Wave III and Wave V latencies, as well as prolonged Wave I–III interpeak intervals, in both ears compared with controls (FDR-adjusted p < 0.05). No significant group differences were observed for Wave V–I interpeak intervals.

### LLR

LLR component latencies and amplitudes are shown in **Table 2**. Latencies of the P1, N1, P2, and N2 components were significantly prolonged in the aphasia group compared with the control group after age adjustment and FDR correction (all FDR-adjusted p < 0.01). No statistically significant group differences were observed for P1–N1 or P2–N2 amplitude measures.

**Figure 1:**
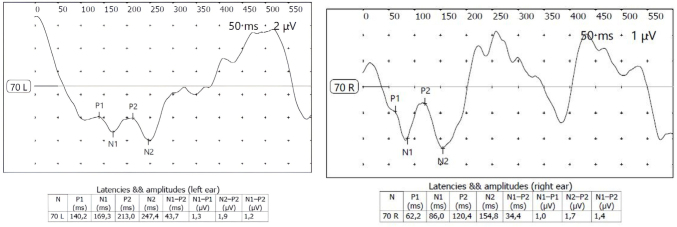
The left panel presents the LLR (Late Latency Response) waveform of a participant from the aphasia group, while the right panel illustrates the LLR waveform of a participant from the control group.

**Figure 1** illustrates representative LLR waveforms from an individual with aphasia and a healthy control participant. Visual inspection demonstrates prolonged P1, N1, P2, and N2 latencies in the aphasia group relative to the control participant, whereas overall waveform morphology remained preserved.

### P300 potentials

**Figure 2:**
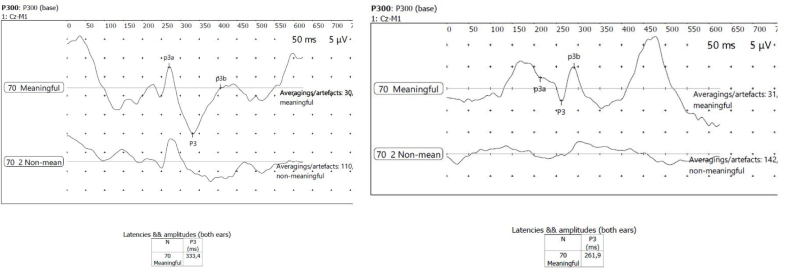
The left panel displays the P300 waveform of a participant from the aphasia group, while the right panel shows the P300 waveform of a participant from the control group.

P300 latency values are presented in **Table 2** and **Figure 2**. The aphasia group exhibited significantly prolonged P300 latencies compared with the control group after controlling for age (F = 15.34, p < 0.001), and this difference remained significant following FDR correction (FDR-adjusted p = 0.001).

**Figure 3:**
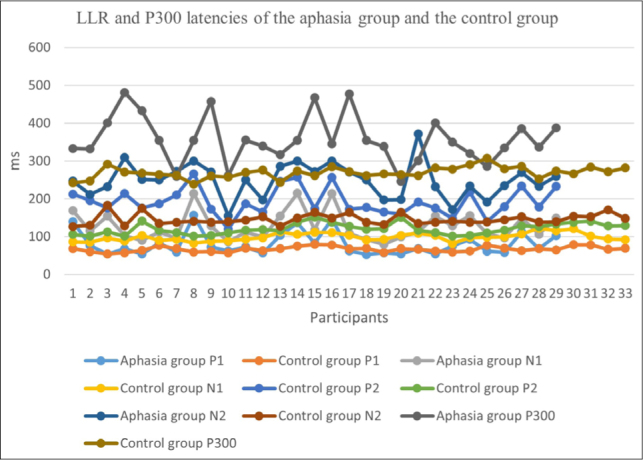
Latency values (ms) of LLR and P300 components in the aphasia and control groups.

**Figure 3** presents group-level comparisons of LLR and P300 latency values across participants. Latency measures for P1, N1, P2, N2, and P300 components were consistently longer in the aphasia group than in controls, reflecting generalized delays in auditory and cognitive processing.

### Correlation between language performance and electrophysiological measures

Partial correlation analyses controlling for age were conducted within the aphasia group. As shown in **Table 3**, moderate negative correlations were observed between naming performance and N2 latency (ρ = −0.324, p = 0.087) and P300 latency (ρ = −0.337, p = 0.073); however, these associations did not reach statistical significance. No significant correlations were observed between language performance and ABR or earlier LLR components (p > 0.05).

**Table 3:** Partial correlations between language performance and electrophysiological measures in the aphasia group (controlled for age)

Measure	ρ	p value
N2 Latency (ms)	−0.324	0.087
P300 Latency (ms)	−0.337	0.073

Overall, latency measures consistently differentiated individuals with aphasia from controls across subcortical, cortical, and cognitive levels, whereas amplitude measures did not.

## DISCUSSION

The present study examined language performance and auditory evoked potentials across multiple neural levels in individuals with post-stroke aphasia. By integrating ABR, LLR, and P300 potentials within the same cohort, this study provides a comprehensive electrophysiological characterization of auditory-cognitive processing in aphasia. The findings demonstrated that individuals with aphasia exhibited significant latency prolongations at both subcortical and cortical levels, whereas amplitude measures remained largely preserved. In parallel, language performance—particularly naming ability—was significantly reduced in the aphasia group. Collectively, these findings suggest that aphasia is associated with generalized slowing in auditory and cognitive processing while neural response magnitude remains preserved.

### Auditory brainstem processing

A key finding of this study was the presence of prolonged ABR wave latencies in individuals with aphasia. Although aphasia is traditionally conceptualized as a cortical language disorder, delayed Waves III and V and prolonged interpeak intervals suggest reduced efficiency in subcortical auditory transmission following stroke. Similar findings were reported by Mourad et al.^6^ in post-stroke aphasia. Nevertheless, ABR findings should be interpreted cautiously because subcortical auditory responses may also be influenced by age-related and vascular factors. Rather than indicating primary peripheral auditory pathology, the observed delays likely reflect altered neural synchrony within the auditory brainstem, which may contribute to degraded auditory input available for higher-level language processing.

### Cortical auditory processing and LLR

At the cortical level, individuals with aphasia demonstrated consistent latency prolongations across all LLR components (P1, N1, P2, and N2), whereas amplitude measures did not differ significantly from controls. This pattern suggests preserved cortical responsiveness with reduced temporal efficiency. Previous studies have similarly reported latency-dominant alterations in cortical auditory evoked potentials in aphasia.^3^ Given the temporal precision required for speech perception and lexical access, such cortical processing delays may interfere with the rapid integration of auditory information necessary for naming and functional communication.

### Auditory–cognitive processing and P300

The most robust electrophysiological difference observed in this study was the marked prolongation of P300 latency in the aphasia group. The P300 component is widely regarded as an index of attention allocation, working memory updating, and stimulus classification.^5^ Prolonged P300 latency therefore reflects slowed cognitive processing rather than diminished attentional capacity. This finding is consistent with previous literature demonstrating delayed P300 responses in aphasia and supports the view that language impairment is embedded within broader auditory–cognitive processing inefficiencies. From a speech and language therapy perspective, these findings underscore the importance of addressing attentional and processing speed components alongside linguistic targets in rehabilitation programs.

### Relationship between language performance and electrophysiological measures

Correlation analyses revealed moderate negative trends between language performance and both N2 and P300 latencies, although these associations did not reach statistical significance. The absence of statistically significant correlations may partly reflect limited statistical power due to the modest sample size rather than a lack of functional association. Nevertheless, the observed trends suggest that delayed cortical and cognitive processing may be functionally relevant to naming performance in aphasia, consistent with prior reports linking cortical auditory evoked potentials to language recovery.^7^ Future studies with larger cohorts are warranted to further clarify these relationships.

### Clinical and translational implications

From a clinical and translational perspective, the present findings highlight the value of latency-based auditory evoked potential measures as objective indices that may complement behavioral language assessments in aphasia.^2^ The preservation of response amplitudes suggests retained neural responsiveness, supporting the potential for rehabilitation-induced plasticity. Integrating auditory-processing and attention-focused interventions into speech and language therapy protocols may therefore improve treatment outcomes. Moreover, electrophysiological measures may provide sensitive tools for monitoring neurophysiological changes during rehabilitation and tailoring individualized intervention strategies.^8^ Such measures may help clinicians identify processing-level limitations not fully captured by behavioral language tests alone.

### Limitations and future directions

Several limitations should be acknowledged. The modest sample size may have limited the detection of statistically significant associations between electrophysiological measures and language performance. Additionally, aphasia subtypes and lesion characteristics were not systematically analyzed, which may have contributed to variability in electrophysiological responses. Future research should incorporate lesion localization, aphasia subtype classification, and longitudinal designs to better elucidate the role of auditory–cognitive processing in language recovery. The lack of statistically significant correlations likely reflects limited statistical power rather than the absence of a functional relationship.

## CONCLUSION

In conclusion, this study demonstrates that post-stroke aphasia is characterized by delayed auditory processing at both subcortical and cortical levels, accompanied by slowed cognitive processing reflected by prolonged P300 latency. These electrophysiological alterations parallel impairments in language performance and support a multilevel auditory–cognitive framework for understanding aphasia. By integrating objective neural measures with behavioral language assessment, this study contributes to the development of more comprehensive and clinically relevant approaches in speech and language therapy.

## Data Availability

Data supporting the findings of this study are available upon request from the corresponding author, Agit Şimşek.
